# Self-Selected Versus Assigned Emotion Regulation in Pain Expectancy: Behavioral and fNIRS Evidence

**DOI:** 10.3390/brainsci16101030

**Published:** 2026-09-26

**Authors:** Qinyi Qiao, Jiahao Du, Fanfu Fang, Ping Shi

**Affiliations:** 1 School of Health Sciences and Engineering, University of Shanghai for Science and Technology, Shanghai 200093, China; 2 Department of Rehabilitation Medicine, Changhai Hospital, Naval Medical University, Shanghai 200433, China

**Keywords:** pain expectancy, emotion regulation, functional near-infrared spectroscopy

## Abstract

**Background:** Expectation is a powerful cognitive modulator of pain, but how different types of emotion regulation approaches interact with expectancy to influence pain processing remains unclear. The present study compared self-selected habitual strategies (cognitive reappraisal or deep breathing) with an assigned non-habitual imagery-based strategy. **Methods:** Twenty-four right-handed healthy participants (11 in the self-selected group, 13 in the assigned strategy group) completed a pain expectancy task under two conditions: Attend (passive observation without regulation) and Regulate (active application of the assigned or self-selected strategy). Electrical stimuli were delivered to the left or right forearm. Functional near-infrared spectroscopy (fNIRS) was used to record prefrontal and sensorimotor cortical activation, and subjective pain ratings were collected using a 0–10 Visual Analogue Scale (VAS). Behavioral ratings, cortical activation (β-values in four regions of interest, ROIs), and neural–behavioral correlations were examined. **Results:** In Attend trials, negative expectancy (high cue followed by medium pain, HM) enhanced pain compared with the no-bias condition (medium cue followed by medium pain, MM), while positive expectancy (low cue followed by medium pain, LM) reduced pain. In Regulate trials, the self-selected group showed a significant LM–MM difference under both left- and right-side stimulation, whereas the assigned strategy group showed a significant LM–MM difference only under left-side stimulation. At the neural level, the Stimulating Location × Group interaction in the left primary somatosensory cortex (L-S1) and right premotor cortex/supplementary motor area (R-PMC/SMA) showed moderate effect sizes ($\eta_{p}^{2}$ = 0.177 and 0.165), but none survived false discovery rate (FDR) correction. In neural–behavioral correlation analyses, one significant correlation was observed in the Attend condition (R-PMC/SMA), along with one marginal trend (L-S1); no significant correlations were found in the Regulate condition after FDR correction. **Conclusions:** Self-selected habitual strategies and assigned non-habitual imagery-based strategies modulate pain expectancy through different behavioral patterns. Habitual self-selected approaches produced more consistent pain reduction under positive expectancy, whereas the assigned imagery-based approach showed weaker and less consistent regulatory effects. However, the neural findings were exploratory and did not survive FDR correction. The advantage of self-selected habitual approaches may lie in self-selection and automaticity rather than in activating specific brain regions. Because stimulation side was not counterbalanced, laterality inferences cannot be drawn.

## 1. Introduction

Pain is not a purely sensory event but a multidimensional experience encompassing sensory-discriminative, affective-motivational, and cognitive-evaluative components that interact with one another [1,2]. This multidimensionality implies that pain perception is substantially modulated by psychological factors—among which expectation, emotion, and cognitive appraisal play particularly critical roles [3].

Among the numerous cognitive factors, expectation—the cognitive process of forming predictions about impending noxious stimuli—has been demonstrated as one of the most powerful modulators of pain experience [4]. The modulatory effects of expectation are achieved through top-down mechanisms involving prefrontal and subcortical structures [5,6]. Recent research has demonstrated that both positive and negative expectations of pain can persist for up to one week, with distinct neural underpinnings: the persistence of positive expectation effects is associated with reduced amygdala activity and enhanced activity in the anterior insula and dorsolateral prefrontal cortex (DLPFC), whereas the persistence of negative expectation effects is predicted by enhanced thalamus activity [7]. Critically, the influence of expectation on pain is intimately intertwined with emotional processing. When individuals anticipate pain, they experience valence-specific emotional responses: negative expectations evoke anxiety, whereas positive expectations reduce the aversiveness of noxious stimuli and elicit pleasant responses [8]. Recent evidence indicates that pain expectation effects are primarily predicted by the emotional responses elicited by expectations rather than by the precision of those expectations [9]. This finding suggests that the emotional component of pain expectancy plays a central role in integrating expectations with sensory inputs. This important role of emotional factors in pain experience accordingly provides a direct theoretical basis for non-pharmacological pain management based on emotion regulation (ER) [10,11]. ER—the processes by which individuals influence which emotions they have, when they have them, and how they experience and express these emotions—serves as a critical bridge between emotional responses to pain expectancy and the subsequent modulation of pain perception [12]. When individuals actively regulate their emotional responses to anticipated pain—for instance, by downregulating cue-elicited anxiety—they can significantly alter pain perception. This capacity is supported by prefrontal regions, including the DLPFC [5,13].

The characteristics of emotion regulation approaches, such as habitual use, self-selection, and automaticity, significantly shape the neural mechanisms of ER by influencing cognitive load, attentional resource allocation, and prefrontal activation efficiency [14,15,16]. Neuroimaging studies have demonstrated that individuals who habitually use cognitive reappraisal exhibit faster behavioral responses and stronger anterior cingulate cortex activity when confronted with negative information, suggesting that habitual strategy use is closely associated with neural efficiency in cognitive control [17]. Imajo et al. further found that self-selected ER strategies produce better stress reduction outcomes than forced-choice strategies, which may be mediated by neural efficiency in the left inferior frontal gyrus [18]. Li et al. also confirmed, using fNIRS, that the efficiency of ventrolateral prefrontal cortex (VLPFC) neurofeedback training is positively correlated with individuals’ habitual use of cognitive reappraisal in daily life, indicating a close relationship between habitual strategy use and activation efficiency in specific prefrontal subregions [19]. From the perspective of cognitive load, familiar techniques—by virtue of established cognitive schemas and automated procedures—require fewer cognitive control resources and achieve higher processing efficiency [20]. Furthermore, different ER strategies have strategy-specific neural signatures: the default mode network provides shared contributions, whereas the amygdala, sensorimotor areas, and frontoparietal control networks provide strategy-specific contributions, implying that when individuals execute unfamiliar strategies, they need to recruit different and potentially non-optimized neural pathways [21]. This implies that when individuals are required to execute non-habitual or assigned techniques that lack automaticity, they may need to recruit different and potentially less efficient neural pathways. When individuals switch between unfamiliar regulatory tactics, lateral frontopolar theta-band activity is significantly enhanced, further indicating that regulatory novelty imposes additional cognitive monitoring load [22]. Nevertheless, most of the above studies have focused on static comparisons between regulatory types, with less attention paid to how the dimensions of “habitual use” and “self-selected vs. Assigned” jointly influence pain expectancy processing.

Despite these advances, several critical gaps remain. Most studies on expectancy-induced pain modulation have focused on the role of expectation itself or emotional responses, with less attention paid to how expectancy processing is differentially modulated when individuals employ different types of emotion regulation approaches—particularly comparing daily habitual self-selected methods (characterized by high familiarity and automaticity) with laboratory-assigned non-habitual techniques (lacking automaticity and not self-selected). The present comparison focuses on two types of regulatory practices that differ in both “degree of habitual use” and “mode of selection”—self-selected habitual approaches formed in daily life versus assigned non-habitual approaches. This comparison more closely approximates the actual regulatory type differences individuals use in clinical and everyday contexts. Moreover, regarding the neural mechanisms of ER, existing evidence is primarily derived from comparisons between strategies such as cognitive reappraisal and expressive suppression, and has not systematically examined whether the distinction between habitual self-selected practices and assigned non-habitual practices engages distinct prefrontal and sensorimotor neural mechanisms. Specifically, whether daily-life habitual regulatory methods (which may include cognitive reappraisal or somatic regulatory techniques such as deep breathing) differ from laboratory-assigned non-habitual approaches in behavioral regulatory effects and neural correlates remains to be elucidated. To address these gaps, the present study employed fNIRS, which was selected for its portability, tolerance to movement, and suitability for measuring cortical hemodynamic responses during pain anticipation tasks, to examine whether behavioral regulatory effects and prefrontal/sensorimotor activation patterns differ between a self-selected habitual regulatory condition (including daily habitual methods such as cognitive reappraisal or deep breathing) and an assigned non-habitual imagery-based regulatory condition during an expectancy-based pain regulation task in right-handed healthy participants [23,24].

## 2. Materials and Methods

### 2.1. Participants

A total of 33 healthy adults participated in the experiment. All participants were right-handed, nonsmokers, and reported normal or corrected-to-normal vision, no red-green color blindness, no chronic or acute illness or pain, no neurological or psychiatric disorders, no pregnancy, no recent use of painkillers, no consumption of illegal drugs, and no participation in another medication- or pain-related experimental study within the past six months. Data from nine participants were excluded: seven due to poor fNIRS signal quality (e.g., excessive motion artifacts, low signal-to-noise ratio) and two due to incomplete data records. This yielded a final sample of 24 participants included in all subsequent analyses.

Given that participants’ emotional states, pain catastrophizing, pain-related anxiety, fear of pain, and baseline affective experience may influence their responses to pain anticipation and actual noxious stimulation, all relevant variables were measured at baseline. Before the experiment, participants completed the Self-Rating Anxiety Scale (SAS), the Pain Catastrophizing Scale (PCS), the Pain Anxiety Symptoms Scale (PASS), the Fear of Pain Questionnaire (FPQ), and the Self-Assessment Manikin (SAM) for affective ratings [25,26,27,28]. Baseline characteristics of participants are summarized in Table 1. Independent-samples t-tests and chi-square tests revealed no significant differences between the two groups in age, sex, height, weight, body mass index (BMI), or any of the psychological questionnaire scores (SAS, PCS, PASS, FPQ, SAM; all *ps* > 0.05). All participants provided written informed consent, and the study was conducted in accordance with the ethical standards laid down in the Declaration of Helsinki.

### 2.2. Experimental Design and Procedure

The experiment consisted of a pre-experimental phase and a formal experimental phase. The pre-experimental phase included pain calibration (on the left side first, followed by the right side), emotion regulation strategy training, and conditioning learning (also on the left side first, followed by the right side). The formal experimental phase consisted of the test session, in which participants completed the pain expectancy task on both the left and right forearms. All participants completed the left side first, followed by a 10-min break after completing the left side, and then the right side. An overview of the experimental procedure is illustrated in Figure 1a.

The pre-experimental phases—pain calibration, strategy training, and conditioning learning—were conducted consecutively, with no rest periods between them. The formal experimental phase followed immediately after the pre-experimental phases without an additional break. Within the formal experimental phase, after completing all trials on the left side, participants received a 10-min rest before proceeding to the right side. The total experimental session lasted approximately 2 h per participant, with variations due to individual differences in strategy training efficiency, conditioning test performance, and fNIRS equipment preparation time.

Participants were not informed of the existence of different strategy groups or their group assignment. The experimenter who conducted the formal test sessions (fNIRS recording, stimulus presentation, and VAS collection) was also unaware of group assignment, as the procedures were identical for both groups. The researcher responsible for strategy training was necessarily aware of group assignment to deliver the appropriate training content; this researcher did not participate in subsequent data collection. The researcher performing statistical analyses used only labels during analysis and was only informed of the correspondence between labels and specific strategy conditions after all analyses were completed. Thus, blinding was maintained for participants, the test session experimenter, and the data analyst, with only the strategy trainer being unblinded due to the nature of the manipulation.

#### 2.2.1. Stimuli

Electrical stimuli were generated by a wireless electrical stimulation system independently developed by the University of Shanghai for Science and Technology. The stimuli were delivered to the medial forearm of the participants through a pair of adhesive surface electrodes (manufactured by Shanghai Litu Medical Equipment Co., Ltd., Shanghai, China; each 4 × 4 cm), which were placed longitudinally on the volar forearm with a center-to-center distance of approximately 6 cm, as shown in Figure 1b. Each stimulus lasted 2 s, with a frequency of 50 Hz and a pulse width of 500 µs. The stimulation current intensity was calibrated individually for each participant prior to the formal experiment.

#### 2.2.2. Group Assignment

Group assignment was based on participants’ self-reported habitual use of emotion regulation strategies. Prior to the experiment, we asked all participants about their daily emotion regulation practices. Participants were asked to describe the specific procedures and contexts in which they used their strategies. Only strategies that had been empirically validated in previous research (e.g., deep breathing, cognitive reappraisal) were accepted. These strategies have been shown to effectively alleviate negative emotional states [29,30,31,32]. Participants who reported using such strategies were assigned to A group (self-selected strategy group); the remaining participants were assigned to B group (assigned strategy group). The final sample comprised 11 participants in A group and 13 participants in B group.

#### 2.2.3. Calibration Session

Before strategy training and conditioning, pain calibration was performed separately for the left and right forearms, in a fixed left-to-right order, to determine three target pain intensities for each stimulation site. Pain intensity was rated using a 0–10 Visual Analogue Scale (VAS), where 0 represented “no pain at all” and 10 represented “unbearable pain” [33,34]. Three target pain levels were defined as 3 (low pain), 5 (medium pain), and 7 (high pain).

The calibration procedure consisted of two phases. In Phase 1 (tolerance threshold estimation), stimulation was delivered starting at 2 mA and increased in 1 mA steps until the participant reported a VAS score of 10, and the corresponding current was recorded as the tolerance threshold. In Phase 2 (precision calibration), stimuli were delivered from 2 mA up to the tolerance threshold, and participants rated each intensity to preliminarily estimate the current values corresponding to VAS 3, 5, and 7. These estimates were then iteratively refined by adjusting the current in 1 mA steps (or with concurrent frequency adjustments) until the participant consistently rated the stimulus at the target pain level on two consecutive trials.

The calibrated stimulation intensities for each participant and stimulation site were recorded for use in the subsequent conditioning and formal experimental sessions.

Independent-samples t-tests were conducted to compare the stimulation intensities between A group and B group. As shown in Table 2, no significant differences were found between the two groups in any of the stimulation intensities (low, medium, or high pain) for either the left or right forearm (all *ps* > 0.05).

#### 2.2.4. Training Session

Following group confirmation and calibration, participants underwent ER strategy training. The experimenter first reconfirmed with each participant the specific content of their assigned strategy—for A group, the operational procedures of their self-selected strategies; for B group, their understanding of the “imagery-based calmness” strategy. Both groups then received standardized training and execution guidance for their respective ER strategies.

A group (self-selected strategy group): Participants completed 3–5 practice trials under the experimenter’s guidance to ensure effective deployment of their self-selected strategies in the pain context.

B group (assigned strategy group): Participants learned the “imagery-based calmness” strategy. The experimenter explained the rationale and provided specific examples (e.g., “blue cue → blue sky”) [8]. Participants then completed 3–5 practice trials to establish associations between different cues and their corresponding calm imagery.

At the end of the training, participants were introduced to the two task instructions that would appear in the formal experiment:

“Attend” trials required participants to try to recall the pain sensation associated with the cue when they saw it, without applying any emotion regulation strategy.

“Regulate” trials required participants to actively apply their assigned ER strategy (self-selected for A group, imagery-based calmness for B group) to regulate their emotional response to the anticipated pain.

Participants were informed that they would receive further practice combining these instructions with specific pain-predictive cues in the subsequent conditioning phase, and that a final test would be administered before the formal experiment began.

#### 2.2.5. Conditioning Session

The conditioning session was conducted separately for the left and right forearms, with the left side completed first, followed by the right side. This phase aimed to establish conditioned associations between neutral picture cues and high-, medium-, and low-pain intensities, with the cue–stimulus correspondences illustrated in Figure 1c. Three different neutral pictures were presented, each corresponding to one pain intensity level (high, medium, or low), followed by a 2 s electrical stimulus at the corresponding intensity.

Following the conditioning phase, participants completed a mock session based on the same trial structure as the formal experiment (Figure 1e) to verify task comprehension and strategy execution before proceeding to the formal experiment.

During the mock session, participants were required to verbally report both the instruction type (“Attend” or “Regulate”) and the expected pain intensity (high/medium/low) associated with the cue for each trial. The test comprised 15 trials, with each of the three cues presented 5 times in random order, and the two instruction types appearing 7 or 8 times respectively.

The criteria for passing the test were as follows:

Correctly identifying the instruction type for each trial;

Accurately recalling the cue–pain association (i.e., reporting the expected pain intensity corresponding to the cue);

Executing the corresponding task according to the instruction (i.e., applying the assigned ER strategy when “Regulate” appeared, and attempting to recall the pain sensation associated with the cue when “Attend” appeared).

Only after the experimenter confirmed that participants met all three criteria were they allowed to proceed to the formal experiment. If a participant failed to meet any of these criteria, the relevant training procedure was repeated before the participant was retested and allowed to proceed to the formal experiment.

#### 2.2.6. Test Session

The formal experiment was conducted separately for the left and right forearms in a fixed order. Participants first completed all 36 trials on the left forearm, followed by a 10-min break, after which the stimulation electrodes were placed on the right forearm for another 36 trials (72 trials in total). The trial composition was identical for the two stimulation sites.

Each side included 18 Attend trials and 18 Regulate trials. The first 6 trials served as a consolidation phase, during which cues remained perfectly paired with corresponding pain intensities (HH, MM, LL, each appearing twice) to reinforce conditioning. The remaining 30 trials constituted the experimental phase. The five cue–stimulus pairing conditions illustrated in Figure 1d were presented in a pseudorandom order: HH (high cue → high stimulus), HM (high cue → medium stimulus), MM (medium cue → medium stimulus), LM (low cue → medium stimulus), and LL (low cue → low stimulus). Each pair condition appeared 6 times (5 conditions × 6 trials = 30 trials).

The present study manipulated the match between pain-predictive cues and the actual delivered pain stimuli to induce different expectation valences. During the conditioning phase, participants established one-to-one associations between the three neutral picture cues and the three pain intensities (HH, MM, LL). In the formal experimental phase, we intentionally introduced mismatched trials. Specifically, the high-pain cue (H) typically signaled high pain; when this cue was followed by a medium-intensity stimulus (HM condition), participants developed negative expectations—that is, they expected pain higher than what they actually experienced. Conversely, the low-pain cue (L) typically signaled low pain; when this cue was followed by a medium-intensity stimulus (LM condition), participants developed positive expectations—that is, they expected pain lower than what they actually experienced. The medium cue followed by a medium stimulus (MM condition) served as a control condition, in which expectations matched actual pain. By comparing VAS ratings and brain activation patterns across HM, MM, and LM conditions, we were able to isolate the independent effects of expectation valence (positive vs. negative vs. no expectation bias) on pain perception.

All visual content (instructions, cue pictures, and rating interfaces) was presented via standardized pre-recorded videos to ensure temporal precision and consistency. The trial sequence (Figure 1e) was as follows: a 1.5 s instruction screen (“Attend” or “Regulate”), followed by a 4 s pain-predictive cue picture, a 3 s black-screen delay, a 2 s electrical stimulus, a 2 s waiting period, and then a 0–10 VAS presented on the screen for participants to complete the rating within 5 s. The trial concluded with a 6-s inter-trial interval (ITI), after which the next trial began automatically.

### 2.3. fNIRS Recording

Raw data were collected using Oxysoft software (v3.5.15, Artinis Medical Systems, Elst, The Netherlands). The fNIRS system used in this experiment consists of 10 transmitters and 8 receivers, with a spacing of 3 cm between all transmitter and receiver pairs. The light source generated by the transmitter in this system is near-infrared light with wavelengths of 752 nm and 841 nm, which is used to detect the time–concentration change data for HbO2 in the test subject, with a sampling rate of 25 Hz.

The probe configuration consisted of 20 measurement channels, covering four regions of interest (ROIs): the left dorsolateral prefrontal cortex (L-DLPFC; channels 6–15), the left primary somatosensory cortex (L-S1; channels 18–20), the left premotor cortex and supplementary motor area (L-PMC/SMA; channels 5, 16, 17), and the right premotor cortex and supplementary motor area (R-PMC/SMA; channels 1–4). The channel configuration is illustrated in Figure 2.

The selection of ROIs was based on neuroanatomical and functional neuroimaging evidence. The DLPFC is a core hub for pain perception and modulation. A recent activation-likelihood estimation (ALE) meta-analysis demonstrated that the DLPFC exhibits no significant laterality effect during pain processing, with highly consistent activation patterns across both hemispheres. Therefore, L-DLPFC recordings can effectively represent bilateral DLPFC activation [35,36]. Regarding S1, pain-related activation occurs not only contralaterally but also ipsilaterally, and ipsilateral S1 activation does not depend on the integrity of the corpus callosum. Given that painful stimuli were delivered to both forearms in the present study, L-S1 recordings were considered sufficient to reflect bilateral somatosensory pain intensity encoding [37,38]. The PMC/SMA is involved in pain anticipation and motor preparation, with bilateral activation patterns; thus, both left and right PMC/SMA were recorded [39].

### 2.4. fNIRS Data Preprocessing and Analysis

The fNIRS data selected for formal analysis includes the “Baseline” section and the “Pain” section. Data preprocessing was performed using the Homer3 toolbox (v3.0.95, The MathWorks, Inc., Natick, MA, USA) in MATLAB R2020a [40]. After manual inspection, channels with poor signal quality were excluded. Automatic pruning was performed using the PruneChannels function (dRange = [1e^−4^, 1e^+4^], SNRthresh = 5, SDrange = [0.0, 45.0]). Raw intensity data were converted to optical density (OD) using the hmrIntensity2OD function. Motion artifacts were detected using the MotionArtifactByChannel function (tMotion = 0.5, tMask = 3.0, STDEVthresh = 50.0, AMPthresh = 5.00) and corrected using wavelet motion correction (turnon = 1, IQR = 1.5). A bandpass filter (0.01–0.2 Hz) was then applied to attenuate low-frequency drifts and high-frequency physiological noise, such as respiration and heartbeat [41]. The corrected optical density data were then converted to oxygenated hemoglobin (HbO) and deoxygenated hemoglobin (HbR) concentration changes using the modified Beer–Lambert law. A final refinement was performed using the MotionCorrectCbsi function. Previous studies have consistently shown that HbO concentration changes reliably reflect local cerebral blood flow and exhibit a superior signal-to-noise ratio compared to HbR; therefore, HbO concentration was selected as the primary measure of task-evoked cortical activation [42].

A block-design general linear model (GLM) approach was implemented using the NIRS-KIT toolbox in MATLAB R2020a [43]. A square-wave regressor modeling the hemodynamic response was constructed, coding the rest period as −1 and the pain stimulation period as +1. The GLM estimated β-values representing the magnitude of HbO concentration change during pain stimulation relative to baseline. Positive β-values indicated task-related cortical activation, whereas negative β-values indicated deactivation. ROI-level β-values were computed by averaging across channels within each anatomical region. It should be noted that the β-values reported here reflect pain-evoked cortical responses modulated by prior expectancy, rather than isolated neural activity associated with expectancy per se, given the slow hemodynamic response and the preceding cue/anticipation period.

### 2.5. Statistical Analysis

Statistical analyses were conducted using SPSS Statistics 27.0. For behavioral VAS data, repeated-measures one-way analysis of variance (ANOVA) was employed. The Shapiro–Wilk test was used to test the normality of all data, and Mauchly’s test of sphericity was applied to repeated-measures data. If the data met the assumption of normality (*p* > 0.05) and the sphericity assumption was not violated (*p* > 0.05), repeated-measures one-way ANOVA was performed for overall comparisons, with Dunnett’s test for post hoc pairwise comparisons. For the comparison of congruent trials (HH, MM, and LL), Tukey’s post hoc tests were applied. If Mauchly’s test of sphericity was significant (*p* < 0.05), the degrees of freedom were corrected using the Greenhouse–Geisser method. If the data did not meet the normality assumption (*p* < 0.05), the Friedman test (non-parametric repeated-measures ANOVA) was used for overall comparisons, followed by Dunn’s multiple comparison test with Bonferroni correction for *p*-values. For all statistical analyses, a significance level of *p* < 0.05 was considered statistically significant.

For fNIRS data, in both Attend and Regulate trials, a 2 (Stimulation Location: left vs. right) × 2 (Expectancy: positive vs. negative) × 2 (Group: A vs. B) mixed-design repeated-measures ANOVA was conducted on the β-values for each ROI. When a significant interaction effect was observed, simple effects analyses were performed to probe the specific patterns of differences across levels of each factor. To control for inflation of the Type I error rate due to multiple comparisons, all simple effects analyses were corrected using the Bonferroni method. To control for multiple comparisons across ROIs, False Discovery Rate (FDR) correction (Benjamini–Hochberg procedure, *q* = 0.05) was applied to the ANOVA *p*-values within each condition.

Correlation analyses between β-values and VAS pain ratings were performed using Spearman’s rank-order correlation coefficient. This non-parametric approach does not require the data to follow a normal distribution, is robust to outliers, and provides a reliable estimate of monotonic associations, which is particularly advantageous given the relatively small sample size of the present study [44]. To control for multiple comparisons across the numerous correlation tests, FDR correction (Benjamini–Hochberg procedure, *q* = 0.05) was applied to the correlation *p*-values within each condition (Attend and Regulate).

All statistical tests were two-tailed. For uncorrected tests, the significance level was set at *p* < 0.05. For FDR-corrected tests, significance was defined at *q* < 0.05.

## 3. Results

### 3.1. Behaviour Data Analysis

#### 3.1.1. VAS Ratings for Congruent Trials (HH, MM, LL) in Attend Trials

To verify the effectiveness of the conditioning procedure—i.e., whether participants successfully established the cue–pain associations—and considering that the Attend condition involves no active emotion regulation, allowing VAS ratings to more purely reflect the cue–pain associations themselves, we analyzed VAS ratings for the congruent trials (HH, MM, LL) exclusively under the Attend condition. A one-way repeated-measures ANOVA with Tukey’s post hoc tests was conducted separately for left- and right-side stimulation.

When the stimulation site was on the left side (Figure 3a), the repeated-measures ANOVA revealed a significant main effect of condition (F(1.849, 46.22) = 353.0, *p* < 0.0001). Post hoc comparisons showed that the HH condition (M = 8.462) produced significantly higher ratings than the MM condition (M = 5.363), with a mean difference of 3.099 (*p* < 0.0001, 95% CI [2.519, 3.679]), and the MM condition produced significantly higher ratings than the LL condition (M = 2.038), with a mean difference of 3.324 (*p* < 0.0001, 95% CI [2.787, 3.862]).

When the stimulation site was on the right side (Figure 3b), a similar pattern emerged (F(1.355, 33.88) = 306.8, *p* < 0.0001). Post hoc comparisons revealed that the HH condition (M = 8.141) produced significantly higher ratings than the MM condition (M = 5.295), with a mean difference of 2.846 (*p* < 0.0001, 95% CI [2.394, 3.298]); the HH condition produced significantly higher ratings than the LL condition (M = 2.162), with a mean difference of 5.980 (*p* < 0.0001, 95% CI [5.200, 6.759]); and the MM condition produced significantly higher ratings than the LL condition, with a mean difference of 3.133 (*p* < 0.0001, 95% CI [2.611, 3.656]).

These results indicate that, regardless of stimulation side, VAS ratings followed a significant HH > MM > LL gradient, confirming that the cue–pain associations established during the conditioning phase were successful. Since the Regulate condition involves active emotion regulation that modulates pain perception, including congruent trials from the Regulate condition in this analysis would confound conditioning effects with regulation effects; therefore, these trials were not included in the present analysis.

#### 3.1.2. VAS Ratings in Attend Trials

In Attend trials, VAS ratings differed significantly across the three pain conditions for both stimulation locations.

When the stimulation site was on the left side (Figure 4a), a repeated-measures ANOVA revealed a significant main effect of condition (F(1.261, 31.53) = 83.69, *p* < 0.0001). Post hoc comparisons showed that the HM condition (M = 6.645) produced significantly higher ratings than the MM condition (M = 5.363, *p* < 0.05), while the LM condition (M = 3.500) produced significantly lower ratings than the MM condition (*p* < 0.05).

When the stimulation site was on the right side (Figure 4b), a similar pattern emerged (F(2.000, 65.23) = 28.86, *p* < 0.0001), with HM (M = 6.577) producing significantly higher ratings than MM (M = 5.295), and LM (M = 3.885) producing significantly lower ratings than MM (all pairwise differences *ps* < 0.05).

#### 3.1.3. VAS Ratings in Regulate Trials

A group: When the stimulation site was on the left side (Figure 5a), the Friedman test revealed significant differences across conditions (χ^2^ = 21.57, *p* < 0.0001). Dunn’s post hoc tests showed that LM was significantly lower than MM (adjusted *p* = 0.0286), whereas HM did not differ from MM (adjusted *p* = 0.0642). When the stimulation site was on the right side (Figure 5b), the Friedman test was also significant (χ^2^ = 9.489, *p* = 0.0087); LM was significantly lower than MM (adjusted *p* = 0.0378), while HM did not differ from MM (adjusted *p* > 0.9999).

B group: When the stimulation site was on the left side (Figure 5c), the one-way repeated-measures ANOVA revealed a significant main effect of condition (F(1.415, 18.40) = 14.60, *p* = 0.0005). Post hoc comparisons using Dunnett’s test (with MM as the control condition) showed that VAS ratings in the HM condition (M = 6.571) were significantly higher than in the MM condition (M = 5.411), with a mean difference of 1.161 (adjusted *p* = 0.0063, 95% CI [0.356, 1.965]), and VAS ratings in the LM condition (M = 4.500) were significantly lower than in the MM condition, with a mean difference of 0.911 (adjusted *p* = 0.0207, 95% CI [0.146, 1.675]). When the stimulation site was on the right side (Figure 5d), the Friedman test was significant (χ^2^ = 22.26, *p* < 0.0001). Dunn’s post hoc tests revealed that HM was significantly higher than MM (adjusted *p* = 0.0280), whereas LM did not differ from MM (adjusted *p* > 0.9999).

### 3.2. fNIRS Processing and Analysis

#### 3.2.1. Attend Trials

In the Attend condition, a 2 (Stimulation Location: left vs. right) × 2 (Expectancy: positive vs. negative) × 2 (Group: A vs. B) mixed-design repeated-measures ANOVA was conducted on the β-values separately for each of the four ROIs. All effects had degrees of freedom of (1, 22). Statistical results are summarized in Table 3.

**Main Effects**

The main effect of Stimulating Location was not significant in L-S1 (F = 2.417, *p* = 0.134, $\eta_{p}^{2}$ = 0.099), L-DLPFC (F = 0.747, *p* = 0.397, $\eta_{p}^{2}$ = 0.033), L-PMC/SMA (F = 0.001, *p* = 0.971, $\eta_{p}^{2}$ = 0.000), or R-PMC/SMA (F = 0.266, *p* = 0.611, $\eta_{p}^{2}$ = 0.012).

The main effect of Expectancy was not significant in L-S1 (F = 0.206, *p* = 0.654, $\eta_{p}^{2}$ = 0.009), L-DLPFC (F = 0.645, *p* = 0.430, $\eta_{p}^{2}$ = 0.028), or L-PMC/SMA (F = 2.473, *p* = 0.130, $\eta_{p}^{2}$ = 0.101); it showed a marginal trend in R-PMC/SMA (F = 4.766, *p* = 0.040, $\eta_{p}^{2}$ = 0.178).

The main effect of Group was not significant in L-S1 (F = 0.502, *p* = 0.486, $\eta_{p}^{2}$ = 0.022), L-DLPFC (F = 0.428, *p* = 0.520, $\eta_{p}^{2}$ = 0.019), L-PMC/SMA (F = 0.859, *p* = 0.364, $\eta_{p}^{2}$ = 0.038), or R-PMC/SMA (F = 0.236, *p* = 0.632, $\eta_{p}^{2}$ = 0.011).

**Interaction Effects**

The Stimulating Location × Expectancy interaction was not significant in L-S1 (F = 1.695, *p* = 0.206, $\eta_{p}^{2}$ = 0.072), L-DLPFC (F = 0.004, *p* = 0.951, $\eta_{p}^{2}$ = 0.000), L-PMC/SMA (F = 0.001, *p* = 0.981, $\eta_{p}^{2}$ = 0.000), or R-PMC/SMA (F = 0.840, *p* = 0.369, $\eta_{p}^{2}$ = 0.037).

The Stimulating Location × Group interaction was not significant in L-S1 (F = 0.901, *p* = 0.353, $\eta_{p}^{2}$ = 0.039), L-DLPFC (F = 0.013, *p* = 0.911, $\eta_{p}^{2}$ = 0.001), L-PMC/SMA (F = 0.083, *p* = 0.777, $\eta_{p}^{2}$ = 0.004), or R-PMC/SMA (F = 0.042, *p* = 0.840, $\eta_{p}^{2}$ = 0.002).

The Expectancy × Group interaction was not significant in L-S1 (F = 0.957, *p* = 0.339, $\eta_{p}^{2}$ = 0.042), L-DLPFC (F = 0.075, *p* = 0.787, $\eta_{p}^{2}$ = 0.003), L-PMC/SMA (F = 0.284, *p* = 0.599, $\eta_{p}^{2}$ = 0.013), or R-PMC/SMA (F = 0.265, *p* = 0.612, $\eta_{p}^{2}$ = 0.012).

The Stimulating Location × Expectancy × Group interaction was not significant in L-S1 (F = 0.113, *p* = 0.740, $\eta_{p}^{2}$ = 0.005), L-DLPFC (F = 1.099, *p* = 0.306, $\eta_{p}^{2}$ = 0.048), or L-PMC/SMA (F = 0.622, *p* = 0.439, $\eta_{p}^{2}$ = 0.027); it showed a marginal trend in R-PMC/SMA (F = 4.480, *p* = 0.046, $\eta_{p}^{2}$ = 0.169).

#### 3.2.2. Regulate Trials

In the Regulate condition, a 2 (Stimulation Location: left vs. right) × 2 (Expectancy: positive vs. negative) × 2 (Group: A vs. B) mixed-design repeated-measures ANOVA was conducted on the β-values separately for each of the four ROIs. All effects had degrees of freedom of (1, 22). Statistical results are summarized in Table 4.

**Main Effects**

The main effect of Stimulating Location was not significant in L-S1 (F = 0.002, *p* = 0.967, $\eta_{p}^{2}$ = 0.000) or L-PMC/SMA (F = 0.030, *p* = 0.864, $\eta_{p}^{2}$ = 0.001); it showed marginal trends in L-DLPFC (F = 3.981, *p* = 0.059, $\eta_{p}^{2}$ = 0.153) and R-PMC/SMA (F = 3.143, *p* = 0.090, $\eta_{p}^{2}$ = 0.125).

The main effect of Expectancy was not significant in L-S1 (F = 0.675, *p* = 0.420, $\eta_{p}^{2}$ = 0.030), L-DLPFC (F = 0.204, *p* = 0.656, $\eta_{p}^{2}$ = 0.009), L-PMC/SMA (F = 0.178, *p* = 0.677, $\eta_{p}^{2}$ = 0.008), or R-PMC/SMA (F = 0.021, *p* = 0.886, $\eta_{p}^{2}$ = 0.001).

The main effect of Group was not significant in L-S1 (F = 0.945, *p* = 0.339, $\eta_{p}^{2}$ = 0.042), L-DLPFC (F = 0.007, *p* = 0.935, $\eta_{p}^{2}$ = 0.000), L-PMC/SMA (F = 0.941, *p* = 0.343, $\eta_{p}^{2}$ = 0.041), or R-PMC/SMA (F = 1.713, *p* = 0.204, $\eta_{p}^{2}$ = 0.072).

**Interaction Effects**

The Stimulating Location × Expectancy interaction was not significant in L-S1 (F = 2.994, *p* = 0.098, $\eta_{p}^{2}$ = 0.120), L-DLPFC (F = 0.896, *p* = 0.354, $\eta_{p}^{2}$ = 0.039), L-PMC/SMA (F = 0.102, *p* = 0.752, $\eta_{p}^{2}$ = 0.005), or R-PMC/SMA (F = 0.224, *p* = 0.641, $\eta_{p}^{2}$ = 0.010).

The Stimulating Location × Group interaction showed marginal trends in L-S1 (F = 4.745, *p* = 0.040, $\eta_{p}^{2}$ = 0.177) and R-PMC/SMA (F = 4.332, *p* = 0.049, $\eta_{p}^{2}$ = 0.165); it was not significant in L-DLPFC (F = 0.902, *p* = 0.353, $\eta_{p}^{2}$ = 0.039) or L-PMC/SMA (F = 0.070, *p* = 0.794, $\eta_{p}^{2}$ = 0.003). FDR correction (Benjamini–Hochberg, *q* = 0.05) was applied to the *p*-values of the Stimulating Location × Group interaction across the four ROIs, and no *p*-value survived the correction.

The Expectancy × Group interaction was not significant in L-S1 (F = 0.703, *p* = 0.411, $\eta_{p}^{2}$ = 0.031), L-DLPFC (F = 0.344, *p* = 0.564, $\eta_{p}^{2}$ = 0.015), L-PMC/SMA (F = 0.527, *p* = 0.475, $\eta_{p}^{2}$ = 0.023), or R-PMC/SMA (F = 2.341, *p* = 0.140, $\eta_{p}^{2}$ = 0.096).

The Stimulating Location × Expectancy × Group interaction was not significant in L-S1 (F = 2.206, *p* = 0.654, $\eta_{p}^{2}$ = 0.009), L-PMC/SMA (F = 0.269, *p* = 0.609, $\eta_{p}^{2}$ = 0.012), or R-PMC/SMA (F = 0.278, *p* = 0.603, $\eta_{p}^{2}$ = 0.012); it showed a marginal trend in L-DLPFC (F = 5.013, *p* = 0.036, $\eta_{p}^{2}$ = 0.186).

### 3.3. Relationship Between Behaviour and fNIRS Data

#### 3.3.1. Correlation Analysis in Attend Trials

In the Attend condition, Spearman correlations were conducted between ROI-level β-values and VAS ratings under HM and LM conditions. FDR correction (Benjamini–Hochberg, *q* = 0.05) was applied to the 16 correlation *p*-values as a single family. Statistical results are summarized in Table 5.

After FDR correction, one correlation reached significance: Right-side stimulation, LM condition, R-PMC/SMA (*r* = 0.613, *p* = 0.002, q = 0.030; Figure 6).

One additional correlation showed a marginal trend that did not survive FDR correction: Left-side stimulation, LM condition, L-S1 (*r* = 0.517, *p* = 0.007, *q* = 0.054), falling just above the corrected threshold (*q* = 0.05).

The remaining 14 correlations did not survive FDR correction (all *qs* > 0.05).

#### 3.3.2. Correlation Analysis in Regulate Trials

In the Regulate condition, Spearman correlations were conducted separately for A group and B group under HM and LM conditions. FDR correction (Benjamini–Hochberg, *q* = 0.05) was applied to the 32 correlation *p*-values as a single family. Statistical results are summarized in Table 6.

After FDR correction, no significant correlations were observed in either group (all q > 0.05). In A group, the smallest uncorrected *p*-values were observed for Left LM, L-PMC/SMA (*r* = −0.6844, *p* = 0.0155, *q* = 0.331) and Left LM, L-S1 (*r* = −0.6643, *p* = 0.0287, *q* = 0.331). In B group, the smallest uncorrected *p*-value was observed for Right HM, L-PMC/SMA (*r* = 0.6061, *p* = 0.0310, *q* = 0.331).

Because no significant correlations were observed in either group after FDR correction, formal group comparisons of correlation coefficients using Fisher’s r-to-z transformation were not conducted.

## 4. Discussion

The present study investigated how two distinct types of emotion regulation approaches modulate pain expectancy processing across behavioral, neural, and neural–behavioral coupling levels. A group was employed their daily habitual strategies (e.g., cognitive reappraisal or deep breathing); B group employed a laboratory-assigned non-habitual imagery-based strategy. Behaviorally, under passive attention (Attend), negative expectancy enhanced pain while positive expectancy reduced it; under active regulation (Regulate), A group exhibited significant and consistent pain reduction under positive expectancy (LM significantly lower than MM on both sides), whereas negative expectancy did not produce significant regulation (HM did not differ from MM on either side); B group showed significant regulation for both HM and LM on the left side, but only HM was significantly higher than MM on the right side while LM was ineffective. Neurally, although the Stimulating Location × Group interaction in L-S1 and R-PMC/SMA showed moderate effect sizes ($\eta_{p}^{2}$ = 0.177 and 0.165, respectively) in the Regulate condition, none survived FDR correction for multiple comparisons. In the neural–behavioral correlation analyses, one significant correlation was observed in the Attend condition (right-side LM, R-PMC/SMA: *r* = 0.613, *q* = 0.030), along with one marginal trend (left-side LM, L-S1: *r* = 0.517, *q* = 0.054); no significant correlations were observed in the Regulate condition after FDR correction in either group. Notably, all Group-related effects in the Attend condition were non-significant (all *ps* > 0.30), confirming that the two groups were neurally equivalent at baseline and ruling out pre-existing group differences as a confound.

### 4.1. Behavioral Modulation Effects of Regulatory Approaches

At the behavioral level, A group exhibited significant and stable pain reduction under positive expectancy (LM) across both stimulation sides (LM significantly lower than MM on both sides), whereas negative expectancy (HM) did not produce significant regulation on either side. This selective regulation pattern may reflect the self-selection advantage of habitual approaches; participants in A group were able to employ coping styles that matched their individual cognitive and emotional response patterns. Imajo et al. demonstrated that self-selected emotion regulation strategies have been shown to produce better stress reduction outcomes than forced-choice approaches [18]. While the present study addresses pain-specific regulation rather than general stress, the parallel suggests that the advantage of self-selection may extend across different regulatory contexts—a possibility that warrants further investigation. Moreover, because A group’s methods were habitually used, their degree of automaticity and execution fluency may have been higher, potentially contributing to their more stable regulatory performance.

B group’s performance was more complex: both HM and LM were significantly regulated on the left side, but only HM was significantly higher than MM on the right side while LM was ineffective. These differential effects across stimulation sides may relate to experimental order confounds (left side first, then right side) influencing regulatory execution proficiency or fatigue/habituation—participants had more task experience on the right side—but the specific mechanisms remain unclear and require further validation in counterbalanced designs. The limitations of B group’s regulatory scheme may stem from its “abstractness” and reliance on mental imagery. Imagery-guided relaxation requires participants to actively construct mental images, a process that imposes high demands on working memory and cognitive control resources. Under the threatening context of pain expectancy, executing non-habitual cognitive operations may further increase cognitive load and reduce regulatory efficiency.

### 4.2. Cortical Activation Patterns in Relation to Previous Findings

Previous studies have characterized S1 as a region capable of exhibiting both contralateral and bilateral responses to nociceptive stimulation [37]. Furthermore, a growing body of literature implicates the sensorimotor network—encompassing S1, the premotor cortex, and the supplementary motor area—in the anticipatory processing of pain. Specifically, activation in these regions during pain expectancy is thought to reflect the modulation of pain perception and the preparation of defensive motor responses [39,45]. This view is further corroborated by fNIRS-based decoding studies, which have identified the R-PMC/SMA as a critical feature for high-accuracy pain classification.

In the present study, the Stimulating Location × Group interaction in L-S1 and R-PMC/SMA under the Regulate condition showed moderate effect sizes ($\eta_{p}^{2}$ = 0.177 and 0.165, respectively), although neither survived FDR correction. These findings should be interpreted as pain-evoked cortical responses modulated by prior expectancy, rather than as isolated expectancy-related neural activity, because the GLM modeled the pain stimulation period as the main regressor and the hemodynamic response is slow relative to the anticipatory period. Nevertheless, their direction aligns with previous findings, providing preliminary evidence for engagement of these sensorimotor regions in strategy-dependent pain modulation. One possible interpretation is that the two regulatory approaches placed different demands on the motor preparation system. However, given the lack of FDR-corrected significance, this interpretation remains speculative and requires confirmation in future studies.

In our study, the L-DLPFC did not demonstrate significant involvement in strategy-dependent pain modulation, which is inconsistent with a large body of literature emphasizing its pivotal role in cognitive reappraisal and top-down emotion regulation. Lorenz et al. demonstrated that DLPFC exerts control over pain perception through top-down modulation and is a key region for cognitive–emotional regulation [5]. Crawford et al., in an activation-likelihood estimation meta-analysis, demonstrated that DLPFC engagement is a shared feature of both pain perception and modulation, with remarkably similar activation patterns across acute pain, chronic pain, and placebo analgesia [35]. Li et al., using fNIRS, also confirmed the active involvement of DLPFC in experimental pain processing and expectancy modulation [6].

In the present study, under the Regulate condition, the Stimulating Location × Group interaction in L-DLPFC was not significant (*p* = 0.353), and the correlations between L-DLPFC activation and VAS ratings did not reach FDR-corrected significance in either group. Several factors may explain this discrepancy. First, the present regulation task involved anticipation of somatic pain, the neural mechanisms of which may differ from the DLPFC activation patterns typically observed in classic cognitive reappraisal studies, where the regulation target is usually emotional pictures or socially relevant stimuli rather than somatic pain [5,35]. Second, neither type of regulatory approach employed in this study maps cleanly onto the cognitive reappraisal paradigm typically associated with DLPFC activation. A group’s methods included somatic-oriented strategies such as deep breathing, which may act more on autonomic and sensorimotor pathways than on prefrontal cognitive control systems; B group’s imagery-based strategy, while cognitive in nature, relies more on visuospatial imagery and attentional regulation than on the verbal–semantic reappraisal processes that strongly recruit DLPFC. Thus, the absence of robust L-DLPFC engagement may partly reflect the nature of the regulatory approaches used, rather than a failure of regulation per se. Third, statistical power limitations may have played a role. With an effective sample size of 24 participants, any real but weak effects in L-DLPFC may have been undetectable.

### 4.3. Dissociation Between Neural and Behavioral Measures

In the neural–behavioral correlation analyses, we observed an interesting dissociation between the Attend and Regulate conditions. In the Attend condition, under LM (positive expectancy), one significant positive correlation emerged after FDR correction (R-PMC/SMA: *r* = 0.613, *q* = 0.030), along with one marginal trend in L-S1 (*r* = 0.517, *q* = 0.054) that did not survive the FDR-adjusted threshold. These findings suggest that, in the absence of regulation, sensorimotor region activation shows a positive association with subjective pain experience under positive expectancy, though the robustness of this coupling varies across regions.

However, in the Regulate condition, no significant correlations were observed in either group after FDR correction. This brain–behavior decoupling during active regulation may reflect the fact that regulatory processes introduce additional sources of variance—when participants actively implement emotion regulation techniques, brain activation reflects not only pain intensity encoding but also regulatory execution, cognitive control, attention allocation, and other processes. The superposition of these additional processes may disrupt the simple linear relationship between sensorimotor regions and pain experience. Additionally, the two types of regulatory approaches may influence pain perception through different neural mechanisms, further increasing the heterogeneity of brain–behavior relationships within groups.

## 5. Limitations

First, the sample size was relatively modest (N = 24), with even smaller effective group sizes for correlation analyses. This limited statistical power likely contributed to the failure to detect significant effects after FDR correction. While FDR correction is appropriate for exploratory neuroimaging analyses, it may be overly conservative under small-sample conditions. Future studies with larger samples are needed to confirm the neural trends observed here.

Second, and most critically, stimulation side was not counterbalanced across participants—all participants received left-side stimulation before right-side stimulation. Consequently, the factor of stimulation side is completely confounded with experimental session order. We have therefore refrained from interpreting any side-related patterns as reflecting hemispheric specialization and have treated stimulation side solely as a repeated-measures factor without drawing lateralization inferences. In addition, some participants who failed the mock session returned to the conditioning phase, meaning they received a higher number of conditioning stimuli than others. This may have introduced potential sensitization effects, which were not specifically modeled in the present study.

Third, ROI coverage was asymmetric. We only acquired fNIRS data from the left DLPFC and left S1 (with bilateral PMC/SMA coverage). This limited spatial coverage cannot capture the complete activation patterns of both hemispheres, particularly the potential contributions of right DLPFC and right S1. Therefore, any attempt to compare left–right hemispheric differences or to infer lateralization effects is not supported by the present data.

Fourth, there is a conceptual confound between familiarity and regulatory type. A group was selected based on a pre-experiment screening interview and employed daily habitual methods; B group employed laboratory-assigned non-habitual techniques. The two groups differ not only in familiarity but also in regulatory content (cognitive reappraisal/deep breathing vs. imagery-based). Thus, the observed group differences cannot be uniquely attributed to familiarity per se; regulatory type—rather than familiarity—may equally or more strongly drive the observed behavioral and neural trends. Future studies with larger samples could stratify participants by specific strategy type (e.g., cognitive reappraisal vs. deep breathing) to better isolate strategy-specific neural signatures.

Fifth, fNIRS technology itself has limited spatial resolution and restricted depth sensitivity due to near-infrared light penetration limits, which may have precluded capturing activity in some subcortical pain-related regions.

Sixth, there are potential confounding effects of breathing techniques on fNIRS signals. In the present study, 4 participants in A group employed deep breathing as their regulatory method. Deep breathing alters respiratory rate and depth, thereby affecting systemic physiological parameters (e.g., heart rate, blood oxygen saturation, CO_2_ partial pressure), which may influence fNIRS signals through systemic hemodynamic fluctuations. We did not simultaneously monitor respiration, heart rate, or peripheral physiological signals, nor did we employ short-distance channels to control for extracerebral components such as scalp blood flow. Therefore, the observed HbO signal differences between A group and B group cannot be directly interpreted as differences in “neural efficiency”; at least part of the observed effects may originate from respiration-related systemic physiological changes rather than cortical neural activity. Future studies should simultaneously acquire respiratory and heart rate signals, or employ short-distance channels to separate extracerebral components.

Seventh, the present experimental paradigm did not include an active control task in the Attend condition, and we cannot fully ensure that participants did not engage in some form of emotion regulation during the Attend trials. Participants were aware that emotion regulation strategies were aimed at controlling pain, which may have introduced response bias and potentially contributed to the lack of difference between HM and MM in some conditions. Future studies should include an active control task (e.g., a neutral task after the cue) to better isolate the effects of regulation.

## 6. Conclusions

The present study used fNIRS to examine how two distinct types of emotion regulation approaches modulate pain expectancy processing. Behaviorally, the habitual self-selected approach (A group) produced bilateral consistent pain reduction under positive expectancy, whereas the assigned non-habitual imagery-based technique (B group) showed different regulatory patterns across stimulation sides. Neurally, although the Stimulating Location × Group interactions in L-S1 and R-PMC/SMA under the Regulate condition showed moderate effect sizes, none survived FDR correction; neural–behavioral correlation analyses revealed one significant correlation in the Attend condition (R-PMC/SMA) and one marginal trend (L-S1), but no significant correlations after correction in the Regulate condition.

The differential effects of regulatory types may be mediated by mechanisms such as the self-selection advantage of habitual approaches, the degree of automaticity, and cognitive load, rather than simply by “activating specific brain regions.” However, due to the conceptual confound between regulatory type and familiarity, asymmetric ROI coverage, lack of counterbalanced stimulation order, and the potential confounding effects of breathing techniques on fNIRS signals, the present study cannot support any inferences regarding hemispheric lateralization, and the neural findings should be considered exploratory and hypothesis-generating rather than confirmatory.

Despite these limitations, this exploratory fNIRS study offers several observations that may inform future research. First, it provides a behavioral distinction between the regulatory effects of habitual self-selected approaches and assigned imagery-based techniques, offering a clear hypothesis for subsequent studies. Second, the moderate effect size trends observed in sensorimotor regions suggest that emotion regulation of somatic pain may engage sensorimotor pathways alongside or instead of prefrontal cognitive control systems.

Future studies should address the limitations of the present design in several respects. First, the lack of counterbalanced stimulation order should be remedied by adopting a balanced design in which half of the participants receive left-side stimulation first and the other half receive right-side stimulation first, thereby disentangling laterality effects from session-order effects. Second, bilateral fNIRS coverage of DLPFC and S1 should be implemented to capture the complete activation patterns of both hemispheres, enabling a more comprehensive assessment of hemispheric contributions. Third, the conceptual confound between regulatory type and familiarity should be resolved by systematically manipulating both dimensions—for example, by training participants on the same strategy to different levels of automaticity, or by comparing different strategy types under matched familiarity conditions. Fourth, simultaneous acquisition of respiratory and heart rate signals, or the use of short-distance channels to control for extracerebral components, should be incorporated in future fNIRS studies to disentangle neural responses from systemic physiological confounds. Fifth, larger sample sizes are needed to ensure adequate statistical power for detecting moderate effects after rigorous multiple comparison correction. Sixth, replication in clinical chronic pain populations is necessary to determine whether the present findings generalize beyond experimental pain in healthy individuals. Addressing these limitations in future work will help clarify the neural and behavioral mechanisms through which different emotion regulation strategies influence pain perception. Seventh, combining fNIRS with fMRI in future studies could provide a more complete picture of pain-related brain activity by including subcortical regions such as the amygdala and thalamus, which cannot be captured by fNIRS alone.

## Figures and Tables

**Figure 1:**
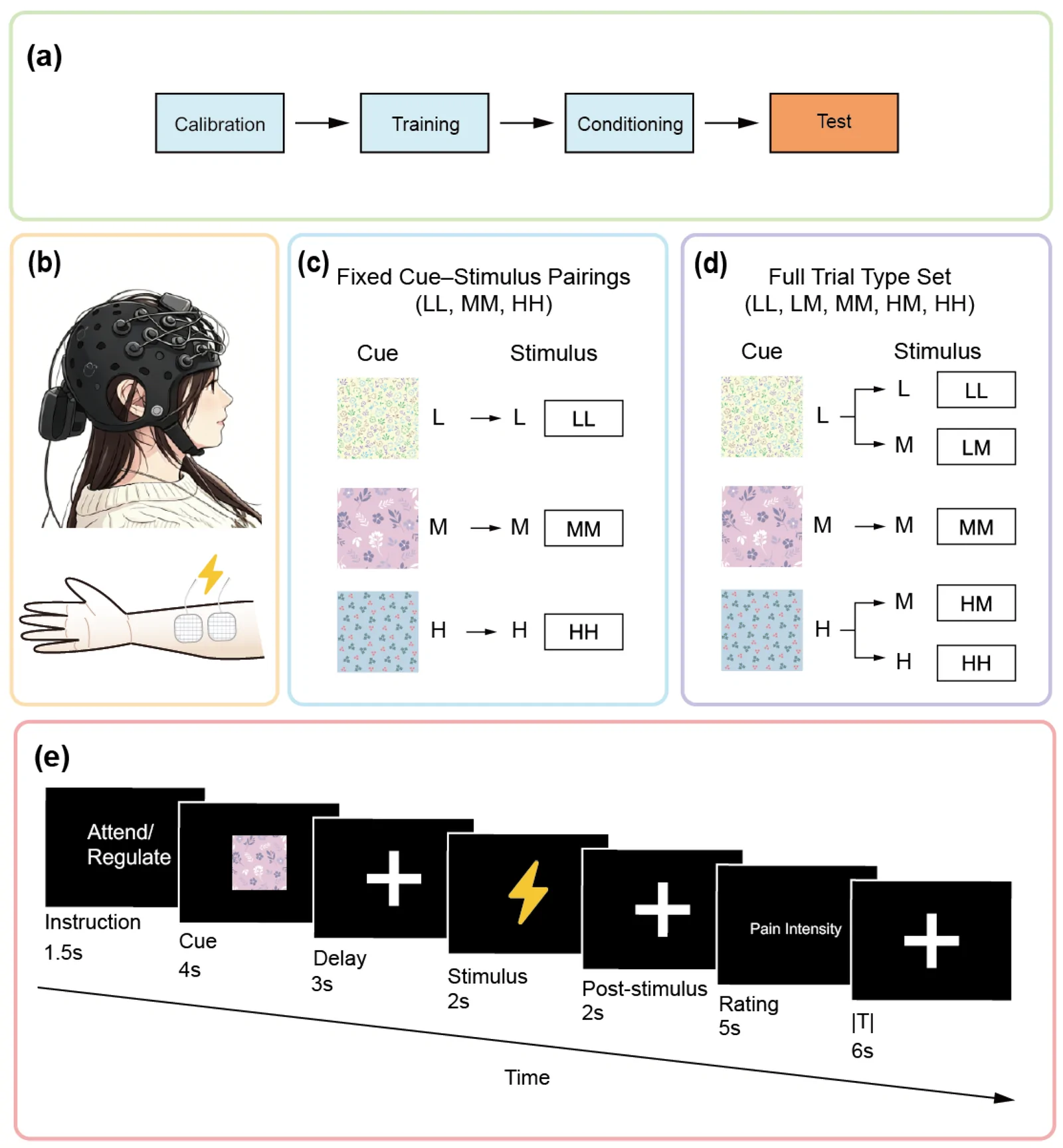
Overview of the experimental design. (**a**) Overview of the pre-experimental and formal experimental procedures. The pre-experimental phase (blue boxes) included pain calibration, emotion regulation strategy training, and conditioning, the formal experimental phase (orange box) consisted of the test session; (**b**) Schematic diagram of the fNIRS device and pain stimulation site, the yellow lightning symbol indicates electrical stimulation; (**c**) Correspondence between picture cues and pain stimulus intensities. During the conditioning phase, three neutral pictures were paired with high, medium, and low pain intensities, respectively (HH, MM, LL); (**d**) Cue–stimulus pairing conditions used in the experimental phase. The experimental phase consisted of five pairing conditions: HH (high cue → high stimulus), HM (high cue → medium stimulus), MM (medium cue → medium stimulus), LM (low cue → medium stimulus), and LL (low cue → low stimulus). The HM and LM conditions were mismatched trials designed to induce negative and positive expectations, respectively; (**e**) Trial structure of the test session. Each trial consisted of a 1.5 s instruction screen (“Attend” or “Regulate”), a 4 s pain-predictive cue, a 3 s delay, a 2 s electrical stimulus, a 2 s waiting period, followed by a VAS pain rating completed within 5 s, with an inter-trial interval of 6 s. fNIRS, functional near-infrared spectroscopy; VAS, Visual Analogue Scale.

**Figure 2:**
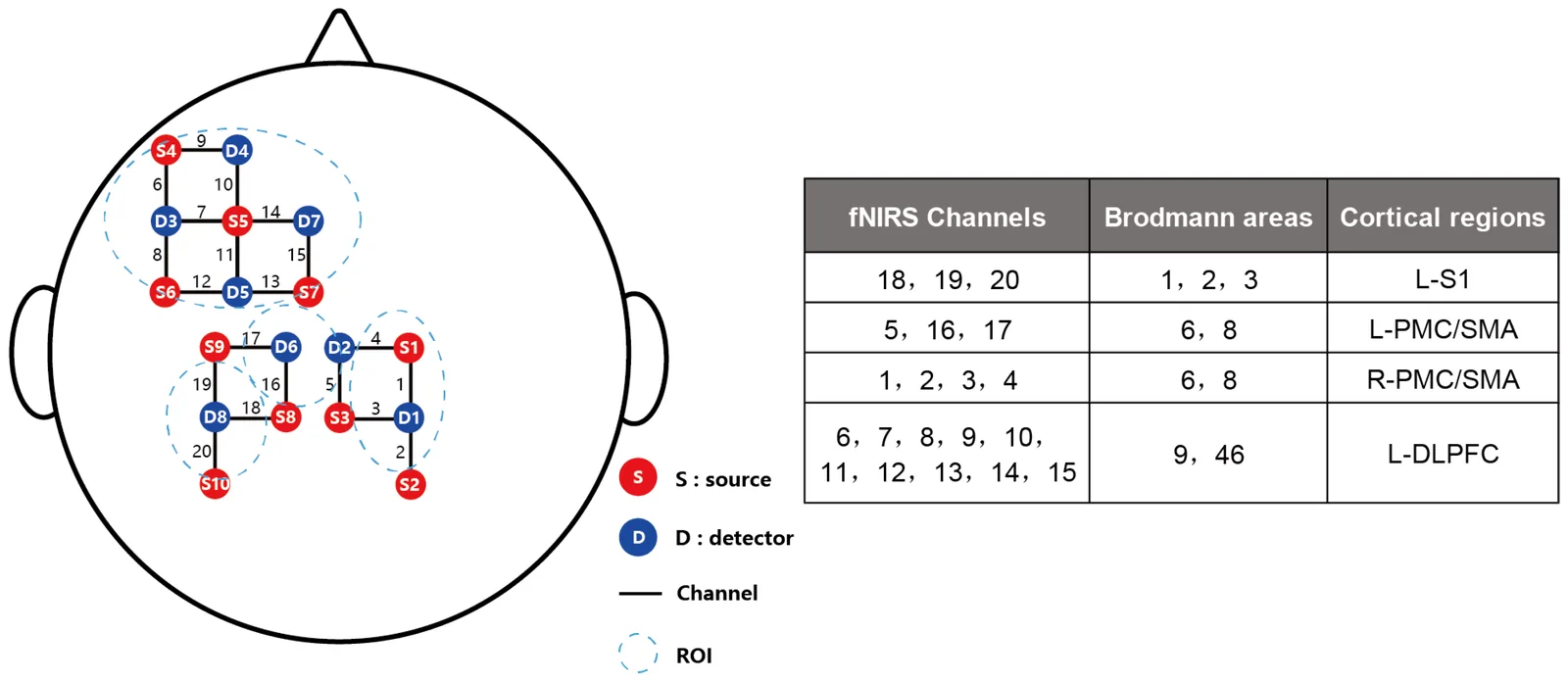
fNIRS probe configuration and channel localization. The red circles represent near-infrared light source fibers, the blue circles represent detectors, and the short black lines represent the measurement channels between source–detector pairs. The blue dashed boxes indicate the channels corresponding to each region of interest (ROI). The table on the right lists the Brodmann areas and corresponding brain regions for each channel. fNIRS, functional near-infrared spectroscopy; DLPFC, dorsolateral prefrontal cortex; S1, primary somatosensory cortex; PMC/SMA, premotor cortex and supplementary motor area.

**Figure 3:**
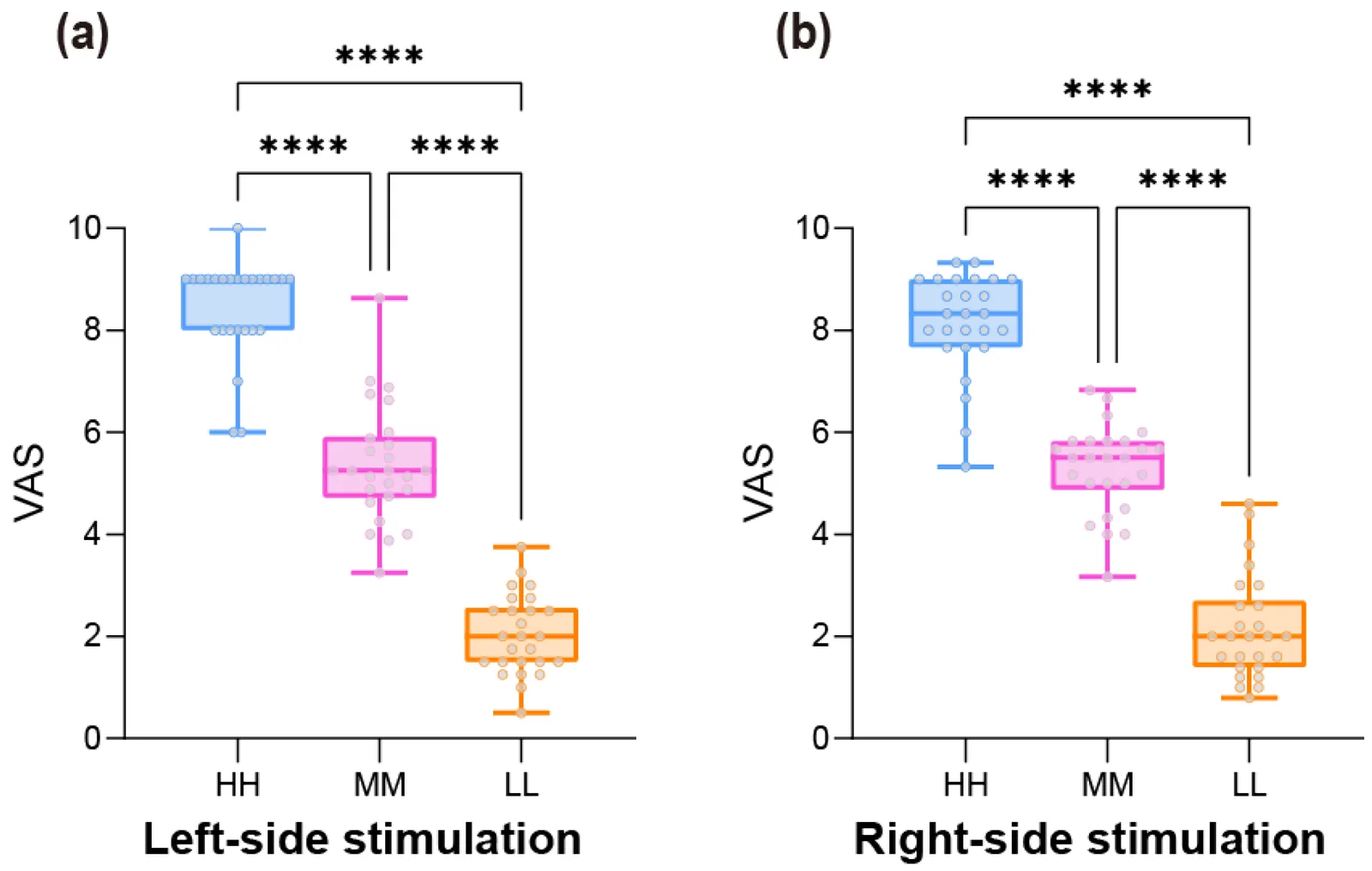
Results of VAS ratings for congruent trials (HH, MM, LL) in Attend trials. Box plots show the median, 25th and 75th quartiles, as well as the 5th and 95th percentiles of VAS ratings for HH (high cue → high stimulus), MM (medium cue → medium stimulus), and LL (low cue → low stimulus) trials under the Attend condition, separately for left-side (**a**) and right-side (**b**) stimulation. HH, MM, and LL are congruent trials used to verify the effectiveness of the conditioning procedure. **** *p* < 0.0001 (one-way repeated-measures ANOVA with Tukey’s post hoc tests). VAS, Visual Analogue Scale.

**Figure 4:**
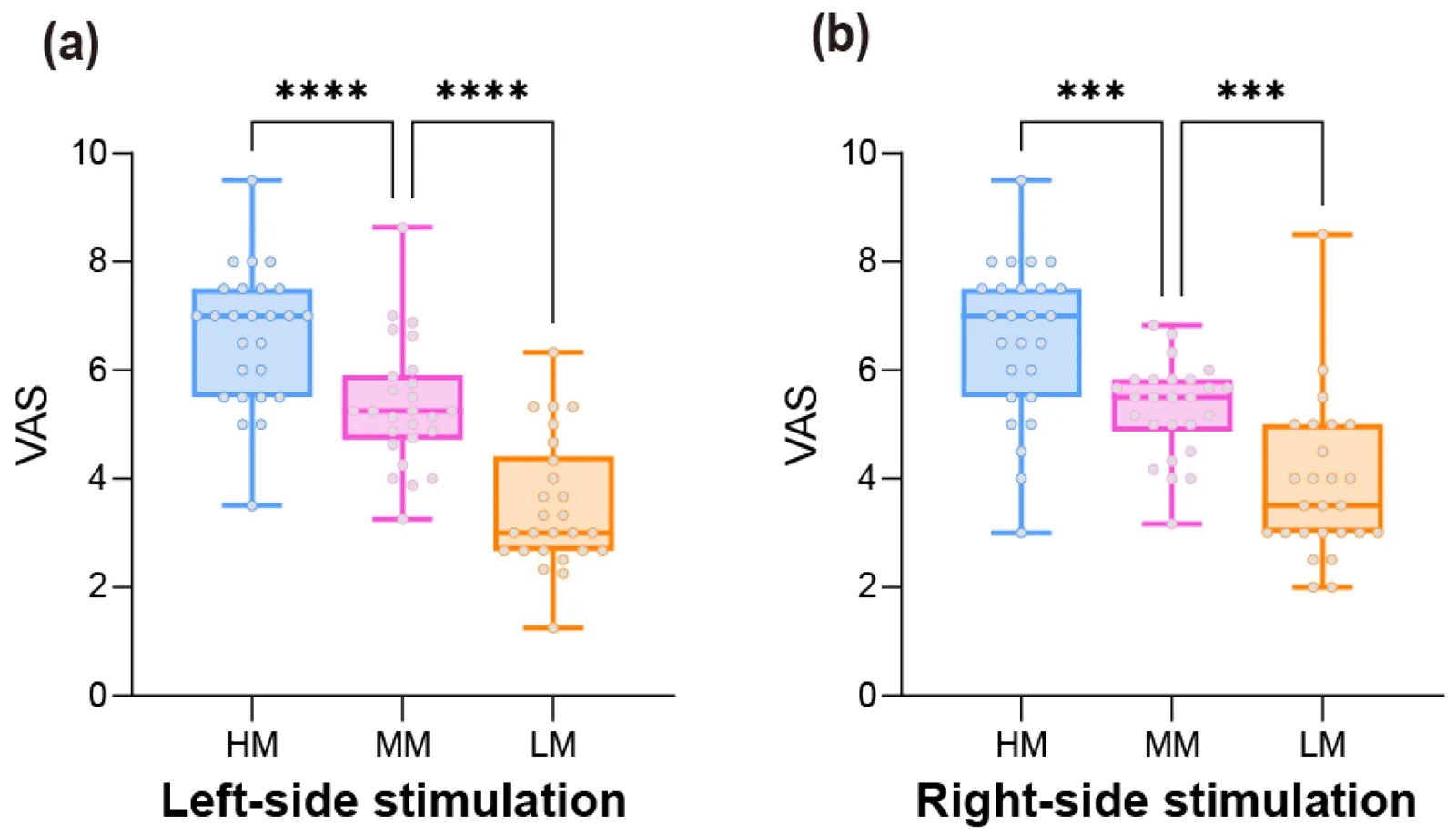
Results of one-way ANOVA for VAS ratings in Attend trials. Box plots show the median, 25th and 75th quartiles, as well as the 5th and 95th percentiles of VAS ratings. *** *p* < 0.001, **** *p* < 0.0001. (**a**) Attend instruction, stimulation on the left side; (**b**) Attend instruction, stimulation on the right side. VAS, Visual Analogue Scale.

**Figure 5:**
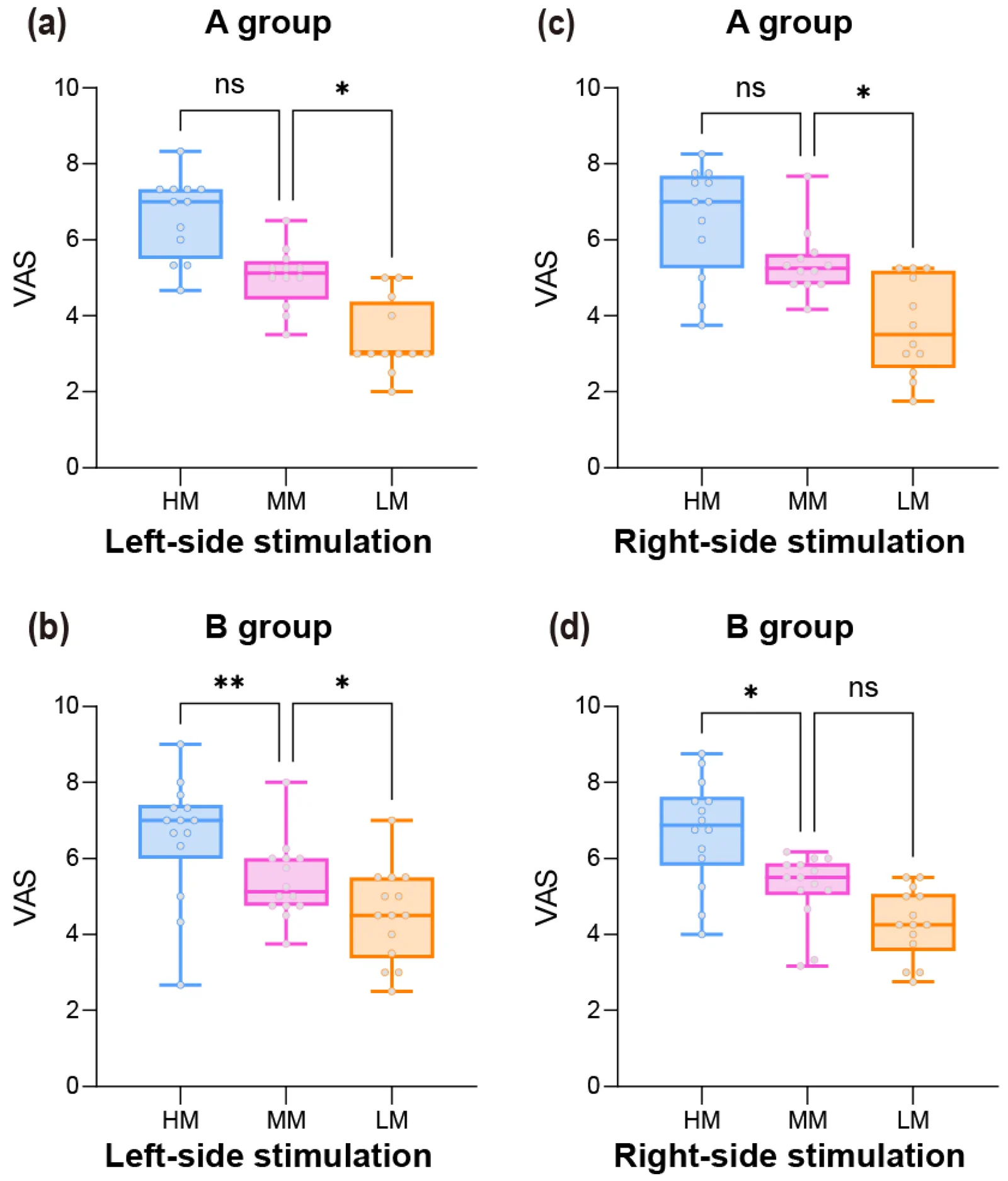
Results of VAS ratings in Regulate trials. Box plots show the median, 25th and 75th quartiles, as well as the 5th and 95th percentiles of VAS ratings in each group and condition. ns indicates non-significance; * *p* < 0.05, ** *p* < 0.01. (**a**) stimulation on the left side, A group; (**b**) stimulation on the right side, A group; (**c**) stimulation on the left side, B group; (**d**) stimulation on the right side, B group. VAS, Visual Analogue Scale.

**Figure 6:**
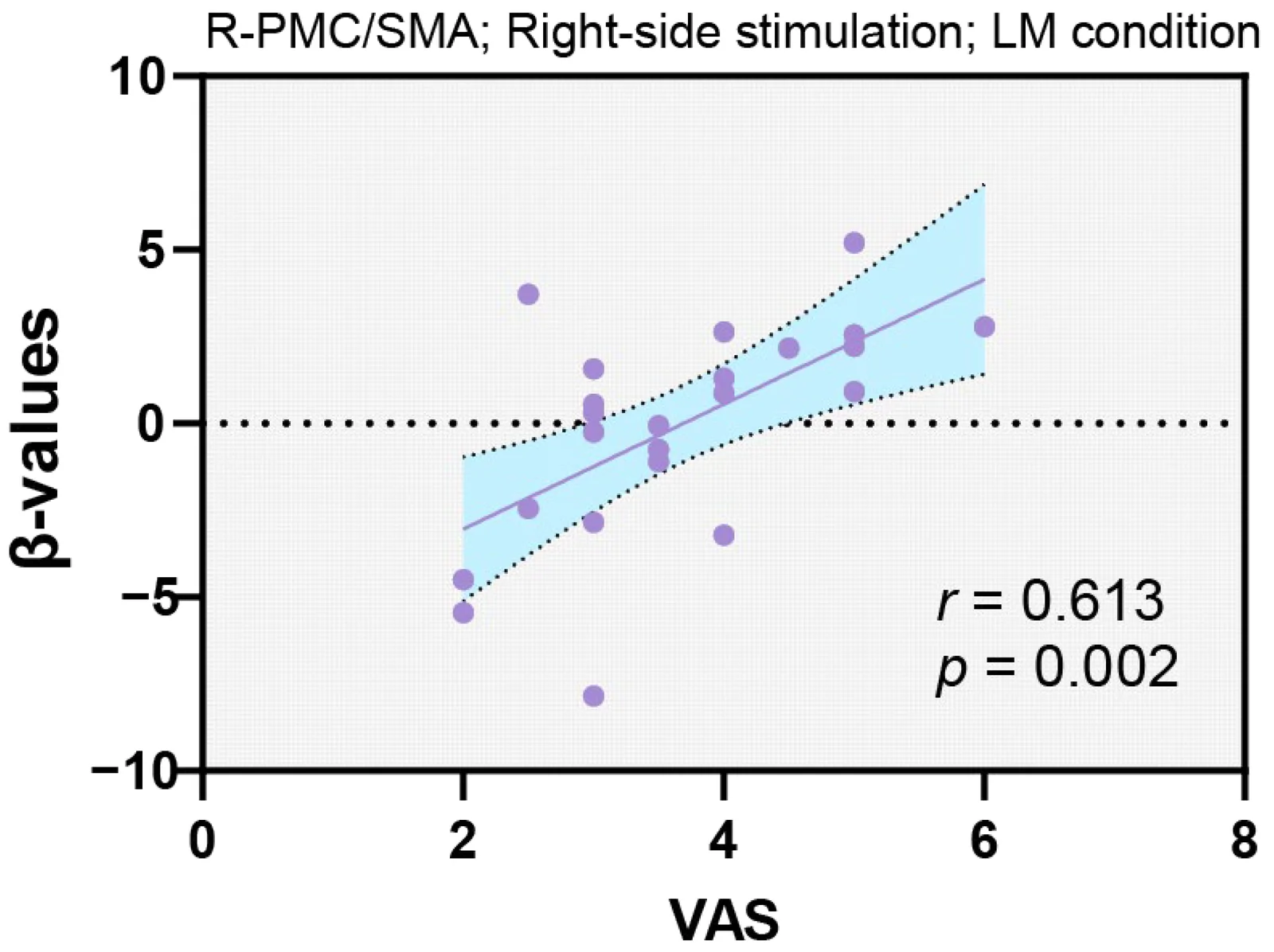
Scatter plot showing the significant correlation between R-PMC/SMA β-value and VAS ratings under the Right-side stimulation, LM condition in Attend trials. The dotted line indicates the linear regression fit, with the shaded area representing the 95% confidence interval.

**Table 1:** Baseline demographic and psychological characteristics of participants by group.

Variable	A Group (*n* = 11)	B Group (*n* = 13)	t/χ^2^	*p*
**Demographics**				
Age	22.18 ± 1.78	23.38 ± 1.56	t = −1.75	0.096
Height (cm)	166.36 ± 5.35	169.15 ± 9.28	t = −0.92	0.370
Weight (kg)	59.27 ± 6.87	65.69 ± 17.94	t = −1.19	0.251
BMI (kg/m^2^)	21.45 ± 2.60	22.64 ± 4.21	t = −0.85	0.406
Gender (male/female)	3/8	8/5	χ^2^ = 1.61	0.205
**Psychological questionnaires**				
SAS	32.82 ± 5.08	31.23 ± 3.27	t = 0.89	0.385
PCS	14.09 ± 8.10	10.92 ± 5.02	t = 1.13	0.277
PASS	60.09 ± 13.01	63.62 ± 10.74	t = −0.72	0.483
FPQ	68.55 ± 14.00	66.31 ± 14.72	t = 0.38	0.707
SAM	6.55 ± 1.13	6.69 ± 1.03	t = −0.33	0.744
SAM	4.09 ± 1.14	4.77 ± 0.93	t = −1.58	0.129

Data are presented as mean ± SD for continuous variables and as n for gender. *p* values were derived from independent-samples *t*-tests for continuous variables and chi-square tests for gender.

**Table 2:** Calibrated electrical stimulation intensities by group.

Variable	A Group (*n* = 11)	B Group (*n* = 13)	t	*p*
**Electrical stimulation intensity (Left side)**				
High pain (H, mA)	9.00 ± 3.44	8.46 ± 3.55	t = 0.38	0.711
Medium pain (M, mA)	5.73 ± 2.05	6.23 ± 3.11	t = −0.47	0.652
Low pain (L, mA)	3.18 ± 1.60	4.15 ± 3.02	t = −1.00	0.349
**Electrical stimulation intensity (Right side)**				
High pain (H, mA)	7.91 ± 3.11	8.23 ± 3.42	t = −0.24	0.813
Medium pain (M, mA)	5.45 ± 2.02	6.23 ± 2.98	t = −0.76	0.471
Low pain (L, mA)	3.64 ± 1.43	4.38 ± 2.81	t = −0.84	0.434

Data are presented as mean ± SD. *p* values were derived from independent-samples *t*-tests.

**Table 3:** Results of repeated-measures ANOVA for each ROI under the Attend condition.

	ROIs			
	L-S1	L-DLPFC	L-PMC/SMA	R-PMC/SMA
**Stimulating location**				
df	(1, 22)	(1, 22)	(1, 22)	(1, 22)
F	2.417	0.747	0.001	0.266
*p*	0.134	0.397	0.971	0.611
$\eta_{p}^{2}$	0.099	0.033	0.000	0.012
**Expectations**				
df	(1, 22)	(1, 22)	(1, 22)	(1, 22)
F	0.206	0.645	2.473	4.766
*p*	0.654	0.430	0.130	0.040
$\eta_{p}^{2}$	0.009	0.028	0.101	0.178
**Group**				
df	(1, 22)	(1, 22)	(1, 22)	(1, 22)
F	0.502	0.428	0.859	0.236
*p*	0.486	0.520	0.364	0.632
$\eta_{p}^{2}$	0.022	0.019	0.038	0.011
Stimulating location * Expectations				
df	(1, 22)	(1, 22)	(1, 22)	(1, 22)
F	1.695	0.004	0.001	0.840
*p*	0.206	0.951	0.981	0.369
$\eta_{p}^{2}$	0.072	0.000	0.000	0.037
Stimulating location * Group				
df	(1, 22)	(1, 22)	(1, 22)	(1, 22)
F	0.901	0.013	0.083	0.042
*p*	0.353	0.911	0.777	0.840
$\eta_{p}^{2}$	0.039	0.001	0.004	0.002
Expectations * Group				
df	(1, 22)	(1, 22)	(1, 22)	(1, 22)
F	0.957	0.075	0.284	0.265
*p*	0.339	0.787	0.599	0.612
$\eta_{p}^{2}$	0.042	0.003	0.013	0.012
Stimulating location * Expectations * Group				
df	(1, 22)	(1, 22)	(1, 22)	(1, 22)
F	0.113	1.099	0.622	4.480
*p*	0.740	0.306	0.439	0.046
$\eta_{p}^{2}$	0.005	0.048	0.027	0.169

**Table 4:** Results of repeated-measures ANOVA for each ROI under the Regulate condition.

	ROIs			
	L-S1	L-DLPFC	L-PMC/SMA	R-PMC/SMA
**Stimulating location**				
df	(1, 22)	(1, 22)	(1, 22)	(1, 22)
F	0.002	3.981	0.030	3.143
*p*	0.967	0.059	0.864	0.090
$\eta_{p}^{2}$	0.000	0.153	0.001	0.125
**Expectations**				
df	(1, 22)	(1, 22)	(1, 22)	(1, 22)
F	0.675	0.204	0.178	0.021
*p*	0.420	0.656	0.677	0.886
$\eta_{p}^{2}$	0.030	0.009	0.008	0.001
**Group**				
df	(1, 22)	(1, 22)	(1, 22)	(1, 22)
F	0.945	0.007	0.941	1.713
*p*	0.339	0.935	0.343	0.204
$\eta_{p}^{2}$	0.042	0.000	0.041	0.072
Stimulating location * Expectations				
df	(1, 22)	(1, 22)	(1, 22)	(1, 22)
F	2.994	0.896	0.102	0.224
*p*	0.098	0.354	0.752	0.641
$\eta_{p}^{2}$	0.120	0.039	0.005	0.010
Stimulating location * Group				
df	(1, 22)	(1, 22)	(1, 22)	(1, 22)
F	4.745	0.902	0.070	4.332
*p*	0.040	0.353	0.794	0.049
$\eta_{p}^{2}$	0.177	0.039	0.003	0.165
Expectations * Group				
df	(1, 22)	(1, 22)	(1, 22)	(1, 22)
F	0.703	0.344	0.527	2.341
*p*	0.411	0.564	0.475	0.140
$\eta_{p}^{2}$	0.031	0.015	0.023	0.096
Stimulating location * Expectations * Group				
df	(1, 22)	(1, 22)	(1, 22)	(1, 22)
F	2.206	5.013	0.269	0.278
*p*	0.654	0.036	0.609	0.603
$\eta_{p}^{2}$	0.009	0.186	0.012	0.12

**Table 5:** Spearman correlations between ROI beta values and VAS ratings in Attend trials.

Stimulating Location	Conditions	ROI	*r*	*p*	*q*
Left	HM	L-S1	0.124	0.547	0.930
		L-DLPFC	0.018	0.930	0.930
		L-PMC/SMA	−0.156	0.447	0.930
		R-PMC/SMA	−0.019	0.926	0.930
	LM	L-S1	0.517	0.007	0.054
		L-DLPFC	0.367	0.065	0.260
		L-PMC/SMA	0.396	0.045	0.241
		R-PMC/SMA	−0.053	0.798	0.930
Right	HM	L-S1	−0.186	0.384	0.930
		L-DLPFC	−0.032	0.883	0.930
		L-PMC/SMA	−0.053	0.807	0.930
		R-PMC/SMA	−0.220	0.301	0.930
	LM	L-S1	−0.040	0.852	0.930
		L-DLPFC	0.041	0.850	0.930
		L-PMC/SMA	0.139	0.518	0.930
		R-PMC/SMA	0.613	0.002	0.030

**Table 6:** Spearman correlations between ROI beta values and VAS ratings in Regulate trials, separately for A group and B group.

Stimulating Location	Conditions	ROI	Group	*r*	*p*	*q*
Left	HM	L-S1	A	0.496	0.103	0.367
			B	−0.215	0.458	0.586
		L-DLPFC	A	0.296	0.348	0.586
			B	−0.106	0.717	0.764
		L-PMC/SMA	A	0.196	0.539	0.615
			B	−0.370	0.193	0.460
		R-PMC/SMA	A	−0.510	0.093	0.367
			B	0.217	0.454	0.586
	LM	L-S1	A	−0.664	0.029	0.331
			B	0.380	0.180	0.460
		L-DLPFC	A	−0.505	0.097	0.367
			B	0.038	0.900	0.900
		L-PMC/SMA	A	−0.684	0.016	0.331
			B	0.247	0.392	0.586
		R-PMC/SMA	A	0.127	0.695	0.764
			B	−0.291	0.310	0.586
Right	HM	L-S1	A	−0.319	0.400	0.586
			B	0.196	0.519	0.615
		L-DLPFC	A	−0.261	0.436	0.586
			B	0.298	0.321	0.586
		L-PMC/SMA	A	−0.500	0.144	0.460
			B	0.606	0.031	0.331
		R-PMC/SMA	A	−0.558	0.078	0.367
			B	0.400	0.176	0.460
	LM	L-S1	A	−0.260	0.436	0.586
			B	−0.379	0.201	0.460
		L-DLPFC	A	−0.626	0.044	0.350
			B	−0.216	0.476	0.586
		L-PMC/SMA	A	−0.101	0.769	0.794
			B	−0.227	0.454	0.586
		R-PMC/SMA	A	−0.534	0.094	0.367
			B	−0.274	0.363	0.586

## Data Availability

The data presented in this study are available on request from the corresponding author due to privacy and ethical restrictions.

## Abbreviations

The following abbreviations are used in this manuscript:

fNIRS	Functional Near-Infrared Spectroscopy
VAS	Visual Analogue Scale
DLPFC	Dorsolateral Prefrontal Cortex
PMC/SMA	Premotor Cortex and Supplementary Motor Area
ER	Emotion Regulation
PFC	Prefrontal Cortex
VLPFC	Ventrolateral Prefrontal Cortex
S1	Primary Somatosensory Cortex
SAS	Self-Rating Anxiety Scale
PCS	Pain Catastrophizing Scale
PASS	Pain Anxiety Symptoms Scale
FPQ	Fear of Pain Questionnaire
SAM	Self-Assessment Manikin
ROI	Region of Interest
ALE	Activation-likelihood estimation
HbO	Oxygenated Hemoglobin
HbR	Deoxygenated Hemoglobin
GLM	General Linear Model
ACC	Anterior Cingulate Cortex
